# Splicing therapeutics for Alzheimer's disease

**DOI:** 10.15252/emmm.201506067

**Published:** 2016-02-22

**Authors:** Catherine R Wasser, Joachim Herz

**Affiliations:** ^1^ Departments of Molecular Genetics, Neuroscience, Neurology and NeurotherapeuticsCenter for Translational Neurodegeneration ResearchUniversity of Texas Southwestern Medical CenterDallasTXUSA

**Keywords:** Neuroscience, Pharmacology & Drug Discovery

## Abstract

The earliest clinical manifestation of Alzheimer's disease (AD) is cognitive impairment caused by synaptic dysfunction. ApoE4, the primary risk factor for late‐onset AD, disrupts synaptic homeostasis by impairing synaptic ApoE receptor trafficking. Alternative splicing of ApoE receptor‐2 (Apoer2) maintains synaptic homeostasis. In this issue, Hinrich *et al* (2016) show that Apoer2 splicing is impaired in human AD brains and murine AD models and that restoring normal splicing in the mouse rescues amyloid‐induced cognitive defects.

The earliest clinical manifestation of Alzheimer's disease (AD) is synaptic dysfunction presenting as memory loss, followed by formation of neuritic plaques neurofibrillary tangles and progressive neurodegeneration. A central component of the trademark plaques is amyloid‐β (Aβ), a product of the amyloid precursor protein (APP). Aβ generation is a by‐product of normal neuronal activity and thus ideally posited to participate in the maintenance of synaptic homeostasis (Sheng *et al*, 2012). In AD brains and to a lesser extent during normal aging, Aβ progressively accumulates, initially in the form of synapse‐suppressing oligomeric complexes, which induce synaptic dysfunction during the early stages of the disease (Fig 1A and B). As agonists of α7‐nicotinic acetylcholine receptors (α7‐NAchR), these Aβ oligomers promote the endocytosis and thus synaptic depletion of NMDA receptors (NMDAR), which are required for long‐term potentiation (LTP) and memory (Snyder *et al*, 2005). Another mechanism by which oligomeric Aβ weakens the synapse is through the activation of metabotropic glutamate receptors (mGluRs), which induce long‐term depression (LTD) through the expression of the phosphatase STEP and dephosphorylation of NMDA receptors on their cytosolic domains. Both mechanisms result in the removal of NMDA receptors from the synapse, and this chronically increasing NMDAR hypofunction leads to the progressive synapse weakening and loss that is responsible for the early cognitive impairment.

**Figure 1:**
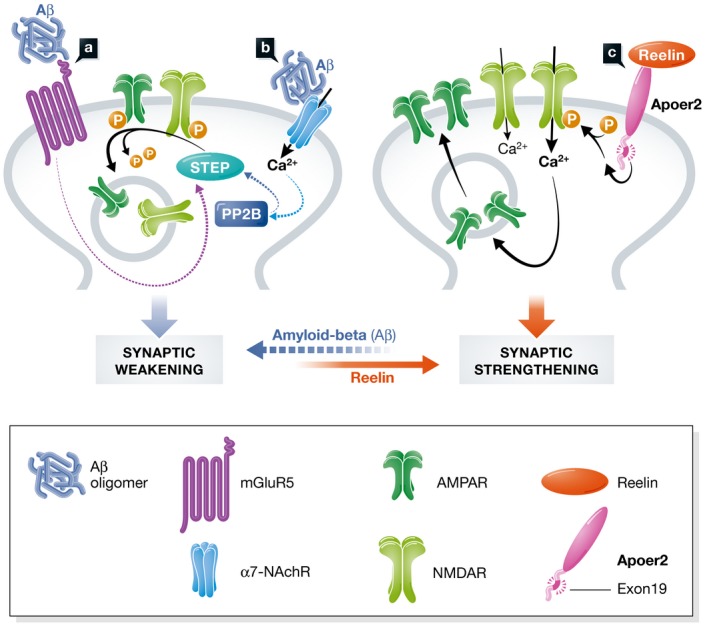
Opposing effects of Reelin and Aβ on synaptic function Aβ interacts with both, metabotropic glutamate receptors, mGluR5 (A), and α7‐nicotinic receptors α7‐nAChRs (B) to promote synaptic weakening (long‐term depression, LTD). mGluR activation by DHPG or Aβ stimulates STEP activity. STEP dephosphorylates AMPARs, promoting receptor endocytosis. Reelin binds Apoer2 (C), which leads to the exon 19‐dependent phosphorylation of NMDARs, enhancing Ca^2+^ influx and enhancing long‐term potentiation (LTP).

The Aβ‐mediated suppression of LTP (and induction of LTD) is opposed by cytoplasmic signaling of an apolipoprotein E (ApoE) receptor, Apoer2, which strengthens the synapse. When activated by the binding of its ligand, the neuromodulator Reelin, postsynaptic Apoer2, together with very low‐density lipoprotein receptor (Vldlr), mediates tyrosine phosphorylation of NMDA receptor NR2 subunits, thereby potently enhancing NMDAR activity and boosting LTP (Fig 1C). This Reelin‐mediated protection against Aβ‐toxicity is selectively impaired by ApoE4, the most prevalent risk factor for late‐onset Alzheimer's disease (Strittmatter & Roses, 1996), which sequesters Apoer2, as well as glutamate receptors that are associated with it, in the recycling endosome. This mechanism suggests that ApoE4 carriers may be more susceptible to the toxic effects of Aβ due to the loss of the synaptoprotective Reelin signal mediated by Apoer2 (reviewed in Lane‐Donovan *et al*, 2014). Understanding the mechanisms underlying Apoer2 function and the regulation of Apoer2 abundance is thus crucial for elucidating the pathogenesis of AD and for exploring new potential therapeutic avenues.

Apoer2 splicing produces a multitude of conserved Apoer2 splice variants, which implies a physiologically significant function for these variants (reviewed in Reddy *et al*, 2011). The ability of a neuron to protect itself against Aβ with Reelin hinges on the alternative splicing of the Apoer2 cytoplasmic domain. The ability of Reelin and Apoer2 to induce the phosphorylation of NMDARs and thus enhance the potentiation of synapses requires the inclusion of a proline‐rich, 59 amino acid insert in the cytoplasmic domain of Apoer2 (encoded by exon 18 in humans and exon 19 in mice). This insert interacts with a postsynaptic scaffolding protein, PSD‐95, which also binds the NR2 subunit of the NMDAR (Beffert *et al*, 2005), and with several other postsynaptic proteins, which regulate synapse formation and neuronal survival (reviewed in Reddy *et al*, 2011).

In a study presented in the current issue, Hinrich *et al* (2016) report that Apoer2 splicing is dysregulated in AD patients, who showed reduced inclusion of exon 18. This would be expected to make them more susceptible to Aβ toxicity. In fact, the cognitive deficits of these patients correlated with the absence of this exon. They extend these findings to present a potential therapeutic approach using a common mouse model of AD, which leads to the robust accumulation of Aβ in plaques as early as 3 months after birth. The authors show that the exon encoding the cytoplasmic insert is also preferentially excluded in these mice, and cerebral injection of antisense oligonucleotides (ASOs) that target the adjacent introns could prevent the exclusion of exon 19. This effect persisted for up to 6 months and proved beneficial to mice with Aβ‐induced cognitive deficits. Hinrich *et al* (2016) further show that ASOs that increase exon 19 inclusion do so by targeting binding sites for a known splicing factor SRSF1, and that the reduction of SRSF1 could also enhance exon 19 inclusion.

Antisense oligonucleotides are a promising therapeutic approach for many human diseases, including neurodegenerative disorders (Choong *et al*, 2015). Splicing is altered in human AD brains (Tollervey *et al*, 2011) across a wide‐range of genes, and so the ability to manipulate the inclusion or exclusion of exons with ASOs is an exciting possibility for treating AD. With no current viable treatments to slow or stop the progression of AD, the findings by Hinrich *et al* (2016) represent an innovative and promising strategy as they suggest that ASOs might also improve cognition in the aging human brain by shifting the splicing of Apoer2 to include this cytoplasmic insert.

The next question to ask is how inclusion of murine exon 19 improves performance on the spatial memory task. The authors propose that increasing the “active” form of Apoer2 is likely enhancing the Reelin‐mediated NMDAR phosphorylation and LTP. Further studies are necessary to determine whether this is in fact the case. Another question is whether this Apoer2 variant is altering Aβ levels. Apoer2 and the Aβ‐precursor, APP, share several common interacting proteins, and Apoer2 can affect APP trafficking and proteolysis (reviewed in Lane‐Donovan *et al*, 2014). However, it is not yet clear whether the presence of exon 19 would lead to decreased Aβ formation, as the authors did not assess Aβ levels in ASO‐treated mice. Reduced Aβ levels, as a consequence of increased exon 19 inclusion, would be expected to further reduce synaptic suppression and enhance cognitive performance.

The study by Hinrich *et al* (2016) provides further evidence that Apoer2 is a critical player in synaptic function and raises the possibility that the ASO technique can be extended to other alternatively spliced exons of Apoer2, specifically a serine/threonine‐rich glycosylation domain (encoded by exon 16). This extracellular sugar domain hinders proteolytic processing of Apoer2, and loss of this sugar domain makes Apoer2 resistant to extracellular proteases. The constitutive expression of cleavage‐resistant Apoer2 *in vivo* augments Apoer2 abundance and dramatically alters both synaptic function and fear memory (Wasser *et al*, 2014). We predict that using the ASO technique to enhance exon 16‐exclusion could also prove cognitively beneficial. By skipping exon 16, Apoer2 abundance would increase and may further counter the Aβ‐mediated synaptic suppression.

Evolution has bestowed on this ApoE receptor a central role in the homeostasis of excitatory synapses by regulating the trafficking of neurotransmitter receptors. To this end, Apoer2 has been endowed with various means of adjusting its own abundance and strength. Alternative splicing of its cytoplasmic domain is such a powerful mechanism.
